# Prediction of Stability during Walking at Simulated Ship’s Rolling Motion Using Accelerometers

**DOI:** 10.3390/s22145416

**Published:** 2022-07-20

**Authors:** Jungyeon Choi, Brian A. Knarr, Yeongjin Gwon, Jong-Hoon Youn

**Affiliations:** 1 College of Information Science and Technology, University of Nebraska at Omaha, Omaha, NE 68182, USA; jungyeonchoi@unomaha.edu; 2 Department of Biomechanics, University of Nebraska at Omaha, Omaha, NE 68182, USA; bknarr@unomaha.edu; 3 Department of Biostatistics, University of Nebraska Medical Center, Omaha, NE 68198, USA; yeongjin.gwon@unmc.edu

**Keywords:** gait stability, ship rolling, center of mass, margin of stability, accelerometer, CAREN

## Abstract

Due to a ship’s extreme motion, there is a risk of injuries and accidents as people may become unbalanced and be injured or fall from the ship. Thus, individuals must adjust their movements when walking in an unstable environment to avoid falling or losing balance. A person’s ability to control their center of mass (COM) during lateral motion is critical to maintaining balance when walking. Dynamic balancing is also crucial to maintain stability while walking. The margin of stability (MOS) is used to define this dynamic balancing. This study aimed to develop a model for predicting balance control and stability in walking on ships by estimating the peak COM excursion and MOS variability using accelerometers. We recruited 30 healthy individuals for this study. During the experiment, participants walked for two minutes at self-selected speeds, and we used a computer-assisted rehabilitation environment (CAREN) system to simulate the roll motion. The proposed prediction models in this study successfully predicted the peak COM excursion and MOS variability. This study may be used to protect and save seafarers or passengers by assessing the risk of balance loss.

## 1. Introduction

Recent advances in wearable sensors have enabled gait analysis outside the laboratory. Continuous gait monitoring during free-living activities presents a promising approach to the gait study, investigating the risk of falling in real-world settings. Individual walking characteristics differ from one individual to another, and walking strategies can change depending on the walking environment [1]. Walking on a moving ship is very different from walking on land. A ship’s movement directly affects a person’s ability to walk [1,2]. The extreme motion of the ship may result in accidents, such as being injured or falling overboard, through the ship causing people to become unbalanced. Such ship’s motion may compromise the safety of sailors and passengers. It was found that 22 people fall off cruise ships each year and only about 20% of them survive [3]. Moreover, according to the National Institute for Occupational Safety and Health (NIOSH), almost one-quarter of all Alaskan fisher deaths between 1990 and 1999 resulted from man overboard (MOB) [4]. Particularly, MOB accidents on a small fishing boat with a few crew members on board are riskier since there is no proper method to alert the MOB condition [5]. Thus, to reduce the likelihood of falling off-board accidents, it is of the utmost importance to predict the risk of falls in the moving environment of a ship.

The human body is less lateral stable when walking [6,7,8,9,10]. The lateral motion control of the center of mass (COM) is essential for maintaining balance during walking [11]. In this regard, the deviation of the gait pattern in the lateral direction has been proposed as a reasonable predictor of falls [11]. The COM excursion was used to assess the control of balance in different ways. Ogaya et al. [12] investigated muscle contributions to the COM excursion during forward body-tilting. Jansen et al. [11] examined how muscle action controls stabilizing mediolateral COM excursion at different walking speeds. Walking in an unstable environment requires individuals to alter their movements to avoid losing balance or falling. Hof and colleagues utilized the velocity of the COM to extrapolate the velocity-controlled position of the COM (XCOM) to demonstrate the mechanical stability of gait [13]. The relationship between the XCOM and the base of support (BOS) indicates the mechanical stability of the system. Dynamic balancing in human walking is essential to maintain stability and can be parameterized by the margin of stability (MOS). Noamani et al. [14] estimated MOS for sitting balance by determining the limit of dynamic stability using wearable device. Based on the Gill et al. study [15], the mediolateral MOS and COM were found to be valid indicators of mediolateral mechanical stability during beam walking. Young and Dingwell [16] found that the MOS variability was affected by wider or longer steps while walking.

Due to ship motion, individuals are subjected to constant perturbations while walking on ships. Since the ship’s length is generally longer than its width, the ship’s movement is usually greater in the roll than in the pitch [17]. For this study, we focused on the roll motion of the ship, which is the primary movement of the ship. In recent studies, persistent perturbations have been used to investigate how non-disabled individuals respond to unstable environments [10,18,19,20,21,22]. The mean and variability of MOS were both increased by continuous lateral perturbations [20]. Accordingly, in a constant perturbation protocol, MOS values can indicate the changes expected to maintain stability under instability conditions [21,22]. These results show that the lateral MOS can be quantified to determine the fall risk. Therefore, if the COM excursion or MOS variability can be predicted using wearable sensors, the risk of falling will be able to be detected during walking during the ship’s rolling motion.

The purpose of this study was to construct a model for predicting balance control and stability in walking on ships by estimating the peak COM excursion and MOS variability. We used the CAREN system during experiments to simulate the roll motion and quantified the peak COM excursion and MOS variability. This study can be used to protect and save seafarers or passengers by determining the risk of falling overboard.

## 2. Materials and Methods

### 2.1. Participants

A total of 30 healthy individuals were recruited for this study. The demographics of the participants are shown in Table 1. All subjects read and signed an informed consent form approved by the University of Nebraska Medical Center Institutional Review Board (IRB 141-21-EP). The general inclusion criterion was being between the ages of 19 and 55 years. Participants were excluded if they had (1) previously had major lower extremity injury or surgery; (2) known cardiovascular conditions that made it unsafe for them to exercise; (3) a history of dizziness due to vestibular disorders, such as Meniere’s disease and vertigo; and (4) any difficulty in walking in unstable moving environments.

### 2.2. Data Collection

**Figure 1:**
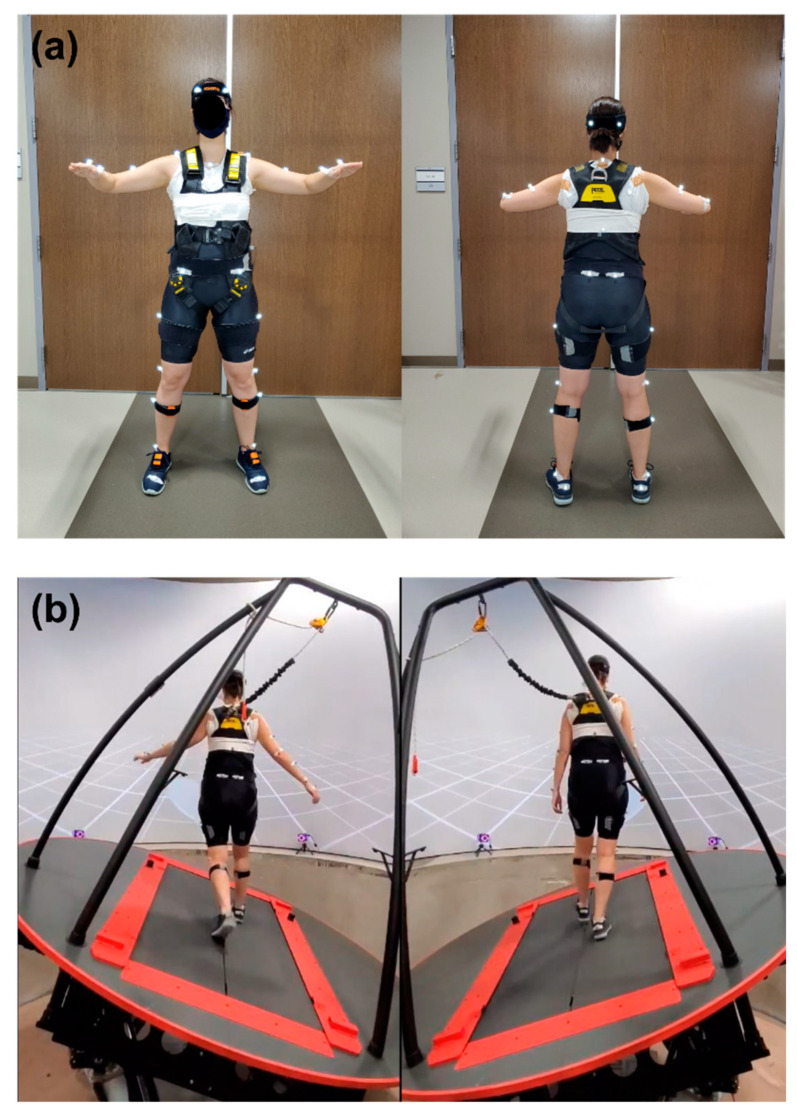
Experimental settings: (**a**) placement of reflective markers and IMU sensors; (**b**) example of simulated roll.

We used a 3D motion capture system (Vicon Motion System Ltd., Oxford, UK) with 10 cameras to record the subjects’ movement at 100 Hz for gold standard data. Thirty-seven reflective markers were attached to anatomical landmarks based on the Plug-in Gait full-body model [23]: four markers on the head, five on the torso, twelve on the upper limb, four on the pelvis, and twelve on the lower limb. We also placed seven accelerometers (Xsens, Enschede, The Netherlands) to obtain three-dimensional accelerations from the pelvis and each foot, shank, and thigh. Since upper body motion is more appropriate to measure balance, the acceleration data from the pelvis were used for data analysis in this study. The placement of reflective makers and accelerometers is shown in Figure 1a. Peak COM excursion was calculated by obtaining the position of COM from the motion captures. MOS variability was calculated by using MOS values from a right limb since there was no significant difference in balance when comparing both sides within participants. To simulate the ship’s roll motion, we used a computer-assisted rehabilitation environment (CAREN) system (Motek, Amsterdam, The Netherlands), simulating up to 20 degrees of rolling.

Participants were asked to walk for two minutes at a self-selected walking speed using the CAREN system with a split-belt treadmill. The simulated roll was tested bilaterally while participants were walking on the CAREN. There were five different conditions: no rolling (NR), 5-, 10-, 15-, and 20-degrees of rolling (i.e., each rolling condition was abbreviated as R5, R10, R15, and R20). Participants performed once for each condition. A safety harness was worn by all participants to avoid accidental falls on the moving platform. Figure 1b illustrates the roll condition while walking on the split-belt treadmill using the CAREN. To prevent learning effects, five different walking trials were conducted in random order.

### 2.3. Step Detection and Feature Extraction

For the step event detection and feature extraction methods, the same methods as in our previous works were used [24,25]. We used a peak detection method for the step detection by recognizing the highest peak of vertical acceleration. Twenty gait features listed in Table 2 were extracted from the pelvis. In addition, the average (denoted by a lowercase “a”), symmetry (denoted by a lowercase “s”), and variability (denoted by a lowercase “v”) of each feature were calculated. A total 60 features were normalized by centering data and then used for this study. Detailed methods for the step detection and feature extraction are well described in [24,25], respectively.

### 2.4. Feature Selection and Modeling

Feature selection is a key part of developing predictive models [26]. The feature selection process involves selecting relevant features and eliminating irrelevant and redundant ones to simplify the model and prevent overfitting. If all possible features are included in a model, overfitting will decrease the model’s performance. It is important to exclude features that are insensitive to sources of variation to avoid overfitting. We examined several feature selection techniques to find the best feature selection method in this study.

#### 2.4.1. LASSO and Elastic Net

The least absolute shrinkage and selection operator (LASSO) minimizes the residual sum of squares of a vector of regression coefficients subject to a constraint on the L1-norm of the vector [27]. This technique is used to estimate and select variables simultaneously, and this method shrinks the coefficients of less important variables to zero, resulting in a sparser model. The LASSO equation is defined as:

(1) $$\overset{n}{\underset{i=1}{\sum }}{\left(y_{i}-\underset{j}{\sum }x_{ij}\beta_{j}\right)}^{2}+\lambda \overset{p}{\underset{j=1}{\sum }}\left|\beta_{j}\right|$$

where *y_i_* and *x_ij_* are the respective outcome and predictors of the *i*th subject; *λ* is a non-negative tuning parameter; and *β* is a vector of regression coefficients that needs to be estimated.

Elastic Net, a combination of ridge regression and LASSO, was proposed in 2005 [28]. When many variables are present and compared to observations, a variable selection based on an elastic net can yield superior results when there is multi-collinearity between predictors [28]. The equation of the elastic net is defined as:

(2) $${min}_{\beta }\left[\frac{1}{n}\overset{n}{\underset{i=1}{\sum }}{\left(y_{i}-x_{l}^{T}\beta \right)}^{2}+\lambda \left(\left(1-\alpha \right)\frac{{\left\|\beta \right\|}_{2}^{2}}{2}+\alpha {\left\|\beta \right\|}_{1}\right)\right]$$

where *y_i_* and $x_{i}^{T}=\left(x_{i1},\cdots ,x_{ip}\right)$ are the respective outcome and predictors of the *i*th subject; *λ* is a non-negative tuning parameter; $\beta ={\left(\beta_{1},\cdots ,\beta_{p}\right)}^{T}$ is a vector of regression coefficients that needs to be estimated; and ${\left\|\beta \right\|}_{1}$ and ${\left\|\beta \right\|}_{2}$ are the regularization terms called *L*_1_*-norm* and *L*_2_*-norm*, respectively:

(3) $${\left\|\beta \right\|}_{1}=\overset{p}{\underset{j=1}{\sum }}\left|\beta_{j}\right|$$

(4) $${\left\|\beta \right\|}_{2}=\sqrt{\overset{p}{\underset{j=1}{\sum }}\beta_{j}^{2}}$$

#### 2.4.2. F-Test Feature Selection

F-tests are used in the feature selection method to test each predictor’s *p*-value individually and rank the features using the *p*-values from the F-tests. The F-test is a statistical procedure used when testing the hypothesis that responses were drawn from populations that have the same mean when comparing it with the alternative hypothesis that the means may not be the same in all populations [29,30]. If the *p*-value of the test statistic is small, the corresponding predictor is significant.

#### 2.4.3. Neighborhood Component Analysis

The neighborhood component analysis (NCA) proposed by Yang et al. [31] is a non-parametric method used to select features for both regression and classification algorithms in order to increase the accuracy of the predictions. This method is ideally suited for the estimation of feature importance for supervised models that are based on pairwise distances between observations to predict responses [31]. Moreover, dimensional reduction using the NCA does not lead to a loss of information [32].

#### 2.4.4. ReliefF Feature Selection

The original ReliefF algorithm [33] estimates the quality of attributes by looking at how well their values distinguish between instances that are close to one another. ReliefF works with a continuous response variable. In this algorithm, predictors that are penalized for assigning different values to neighbors with the same response values are rewarded for assigning different values to neighbors with different response values [34,35]. However, ReliefF computes the final predictor weights based on intermediate weights. ReliefF has the unique ability to exploit information locally while taking the context into account, yet still provide a global perspective [34].

#### 2.4.5. Model Fitting

For fitting the predictive model, we used a linear regression model and a ridge regression depending on the presence of multicollinearity. If there was multicollinearity among the features selected by each feature selection method, the ridge regression model was used; otherwise, we used the linear regression model. The variance inflation factor (VIF) was used to determine the existence of multicollinearity [36]. A linear regression model is commonly used to investigate the relationship between continuous outcome (i.e., peak COM excursion or MOS variability) and independent variables (i.e., selected features) [37]. Ridge regression minimizes the MSE of the estimates by shrinking its coefficients toward zero [38]. This is a regularization method for analyzing all data resulting from the multicollinearity issue [39].

### 2.5. Performance Criteria

To compare the predictive accuracy for our best models constructed by using the different feature selection methods, the mean absolute error (MAE) as a performance measure was calculated for the test data for each model:

(5) $$\mathrm{MAE}=\frac{1}{n}\overset{n}{\underset{i=1}{\sum }}\left|Yp\left(i\right)-Ya\left(i\right)\right|$$

where $Y_{p}\left(i\right)$ and $Y_{a}\left(i\right)$ are the respective predicted and actual values of the *i*th subject for each response variable.

The performance of our model was evaluated using the following criteria. First, we split the whole dataset into a ratio of 7 to 3 for training and testing datasets, respectively. The regression coefficients were determined by the training set. These coefficients were then used to predict the COM excursion and MOS variability for the testing set. This process was repeated 100 times using a random selection of training and testing datasets for each iteration. In all comparisons, each model for the different selection methods was executed using the same set of random selections, ensuring that the validation dataset was the same across models.

### 2.6. Statistical Analysis

A paired *t*-test was used to determine the mean difference between the actual values and the predicted values for peak COM excursion and MOS variability. We assumed that if there was no significant difference between the actual and predicted values, the prediction results were reliable. In addition to the *p*-value approach, we also examined meaningful change in the peak COM excursion and MOS variability so we could compare our prediction results to the actual values using an effect size. Effect size quantifies a difference between two means based on distribution so that the results of different measures can be compared. The effect size is calculated using Cohen’s d, which is defined as [40]:

(6) $$d=\frac{\left(\mu_{1}-\mu_{2}\right)}{\sigma_{1}}$$

where *u*_1_ and *u*_2_, respectively, are the means of actual values and predicted values and $\sigma_{1}$ is the standard deviation of actual values. For interpreting the effect size, the values of <0.2, 0.5–0.6, and >0.8 represent small, medium, and large changes, respectively [40]. All statistical analyses were performed using MATLAB version R2020a (Mathworks Inc., Natick, MA, USA) and statistical significance was set at *p* < 0.05.

## 3. Results

### 3.1. Feature Selection and Model Fitting Results

We initially used five different feature selection algorithms. Elastic Net and NCA methods were excluded because the selected features varied according to data of different scales. Accordingly, we compared only the remaining three methods, LASSO, F-test, and ReliefF, to achieve consistent results regardless of the data scale. The top 10 most selected features with the three methods are shown in Table 3. For peak COM excursion, vAHS and sAHS were commonly selected in three methods and similar features were selected between LASSO and ReliefF. For MOS variability, there were no features selected commonly in the three methods and similar features were selected between LASSO and ReliefF. We observed more consistency in feature selection between LASSO and ReliefF than between the F-test and either of the other two methods. The initial 10% of step-related features (e.g., AHS, LHS, LHM, VHS, and VHM) and double-stance-related (e.g., AMD) features were mostly selected in three methods for both peak COM excursion and MOS variability. The variability-related features were most selected with the F-test method for both dependent variables.

To fit the predictive model, we first checked VIF values to see if there was multicollinearity among the top 10 features selected by three feature selection methods. If the VIF was greater than 5, the features were highly correlated [36,41]. The VIF values between the selected features with three different feature selection methods for both dependent variables are represented in Table 4. Based on the results, we found that LASSO has no multicollinearity while the F-test and ReliefF have multicollinearity problems in peak COM excursion and MOS variability. Thus, a linear regression model was used for the LASSO method, and a ridge regression model was used for the F-test and ReliefF methods.

### 3.2. Prediction and Validation Results

We calculated the MAE for each model summarized in Table 5 and then compared the results to choose the best model. The best models were selected with the most petite MAE: the top seven features with LASSO for peak COM excursion (MAE: 0.0883) and the top ten features with the LASSO for MS variability (MAE: 0.0041).

**Figure 2:**
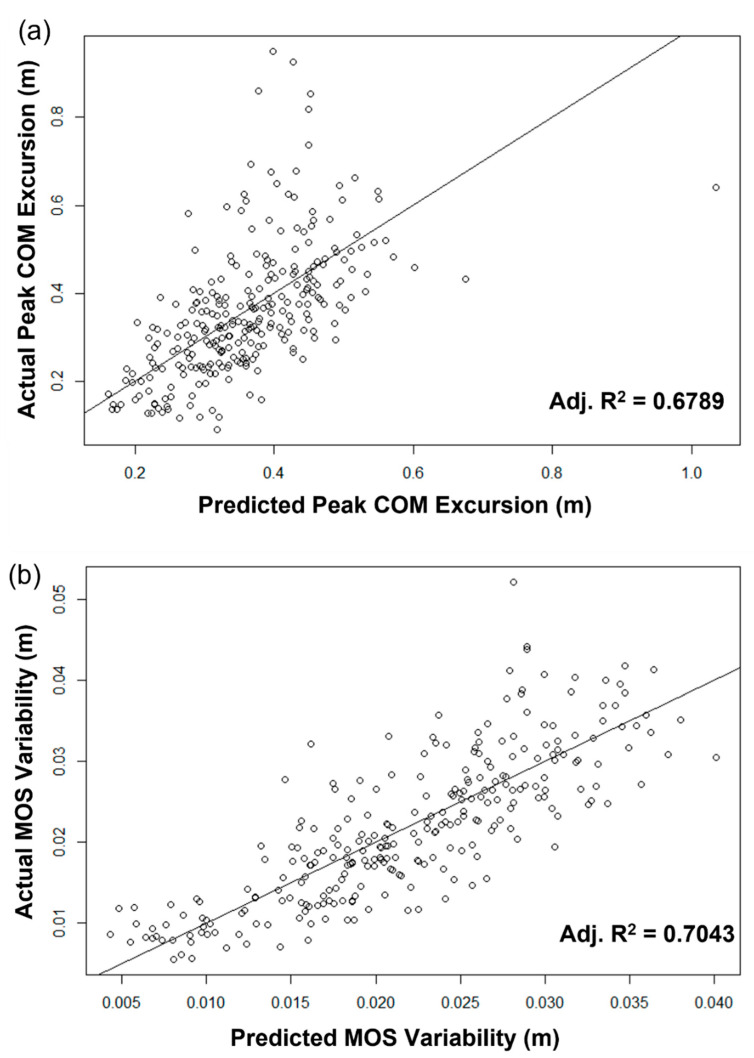
Scatter plots for predicted results vs. actual values: (**a**) peak COM excursion and (**b**) MOS variability.

The prediction results for the selected best models are shown in Figure 2. For comparison of the best models among different feature selection methods, the LASSO performed better than others (MAEs for peak COM excursion: LASSO (0.0883 m) > F-test (0.0896 m) > ReliefF (0.0909 m), and for MOS variability: LASSO (0.0041 m) > ReliefF (0.00466 m) > F-test (0.0051 m)).

**Figure 3:**
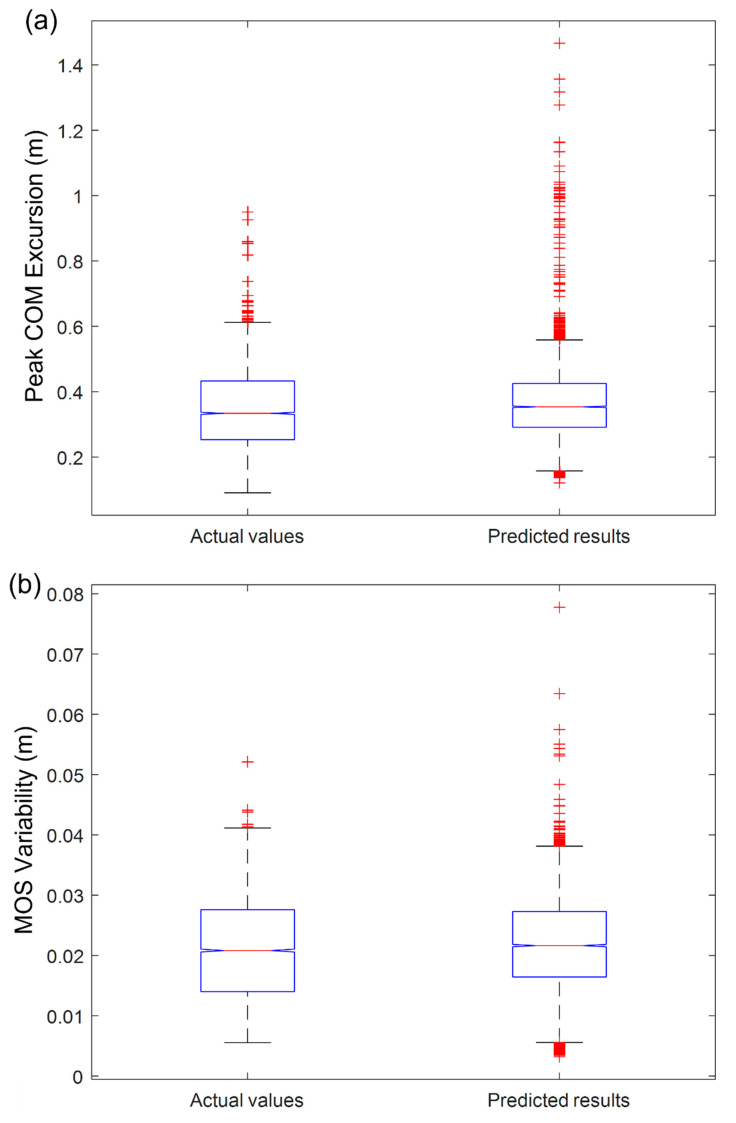
Comparison of the differences between actual values and predicted results for: (**a**) peak COM excursion and (**b**) MOS variability.

To validate our prediction model, we performed a paired *t*-test between actual value and predicted value. Comparisons between the predicted results and the actual measured values for peak COM excursion and MOS variability by the paired *t*-test are shown in Figure 3 and Table 6. There was no difference between the actual and predicted values for the peak COM excursion (*p* = 0.0527) while there were significant differences between the actual and predicted values for the MOS variability (*p* = 0.0318). For determining the practical significance, we also computed the effect size using Cohen’s *d*. The effect sizes for the peak COM excursion and MOS variability were 0.0053 and 0.0111, respectively.

## 4. Discussion

This study demonstrates that wearable sensors can be used to predict gait stability on a ship in simulated sea conditions. Utilizing the best feature selection method and linear regression models, we developed prediction models for peak COM excursion and MOS variability. Intuitively, the prediction errors were minor, and the adjusted r-squared values of the prediction models for the peak COM excursion and MOS variability look reliable at 0.6789 and 0.7043, respectively (Figure 2). We employed paired t-test analysis to evaluate the reliability of the developed models. As shown in Table 6 and Figure 3, we found no difference in the peak COM excursion (*p* = 0.0527), which means our prediction result for the peak COM excursion was reliable. On the other hand, there was a statistically significant difference in MOS variability (*p* = 0.0318) at the 95% significance level, but we can say that there was no difference at the 90% significance level. In addition, we used an effect size to determine the practical significance of our research results. The effect size indicates the importance of the difference between groups. Statistical significance using the *p*-value can be deceptive as it is affected by the large sample size [40]. The effect sizes between the actual values and the prediction results for the COM excursion and MOS variability were 0.0053 and 0.0111, respectively (Table 6). The effect sizes were less than 0.2, which means there were no practical differences in both variables. Thus, we proved that our prediction results were reliable.

Furthermore, the study exhibited the best feature selection method for predicting the peak COM excursion and MOS variability. The results of our research indicated that the LASSO gave the best prediction results with the smallest MAE (Table 5). The best MAEs with the LASSO for predicting the peak COM excursion and MOS variability were 0.0883 m and 0.0041 m, respectively. Previous studies have quantified the lateral COM and MOS to determine the fall risk [10,18,19,20,21,22]. Therefore, we can predict fall risk while walking in sea environments by estimating these variables.

There are several limitations to this study. First, the participants are relatively young and healthy individuals and have little experience onboard a ship. Therefore, it is unreasonable to generalize our results to experienced sailors and middle-aged and older cruise ships’ main customers. Nevertheless, our findings are sufficient to predict the walking stability of young and inexperienced trainees or new crew members because they are more likely to lose balance with ship movements than experienced crew members. Second, only the ship’s rolling motion was applied in the experiment. The actual movement of the ship in the sea involves six degrees of freedom, including rolling, pitching, etc. In addition, the actual ship has a rolling motion of more than 20 degrees in bad weather, but only 20 degrees of rolling were tested in our experiment since the CAREN system only supports up to 20 degrees. However, this was the first study to predict walking stability in a sea environment to the best of our knowledge. Therefore, further research is needed for verification by applying our method to ships in real-world sea environments. Lastly, the predictions of COM excursion and MOS variability may be affected by individual differences, such as age, height, weight, BMI, or their balance control ability. In the experimental design of future studies, therefore, these human factors should be taken into account in order to examine individuals’ differences.

## 5. Conclusions

This study investigated whether typical dynamic stability measures, peak COM excursion, and MOS variability could be predicted in healthy individuals walking in sea environments using wearable sensors. The proposed prediction models in this study successfully predicted the peak COM excursion and MOS variability. We also assessed three feature selection methods for predicting gait stability on a ship at sea by estimating the peak COM excursion and MOS variability. The LASSO resulted in the lowest prediction errors. Our findings can be used to assess the risk of balance loss. Further studies should investigate the validity of these findings when the methods are applied to a real sea environment to prevent falling overboard by detecting the risk of falls.

## Figures and Tables

**Table 1:** Participants’ demographics.

Characteristics	Mean ± Standard Deviation
Gender (male/female)	20/10
Age (years)	30.3 ± 6.1
Height (cm)	173.0 ± 9.4
Weight (kg)	71.9 ± 14.5
Body Mass Index (BMI) (kg/m^2^)	23.8 ± 3.4

**Table 2:** Description of extracted features.

Feature	Description
M	Whole step vector magnitude
M10	Initial 10% step vector magnitude
LM	Lateral vector magnitude during a whole step
VM	Vertical vector magnitude during a whole step
AM	Anterior–posterior vector magnitude during a whole step
MD	Vector magnitude during double stance
LMD	Lateral vector magnitude during double stance
VMD	Vertical vector magnitude during double stance
AMD	Anterior-posterior vector magnitude during double stance
M30	Vector magnitude during mid-stance
LM30	Lateral vector magnitude during mid-stance
VM30	Vertical vector magnitude during mid-stance
AM30	Anterior–posterior vector magnitude during mid-stance
LHM	Lateral heel-strike magnitude
LHS	Standard deviation of lateral acceleration during initial 10% step
VHM	Vertical heel-strike magnitude
VHS	Standard deviation of vertical acceleration during initial 10% step
AHM	Anterior–posterior heel-strike magnitude
AHS	Standard deviation of anterior-posterior acceleration during initial 10% step
ST	Step Time

**Table 3:** Top 10 selected features for peak COM excursion and MOS variability using LASSO, F-test, and ReliefF (* indicates that rank is the same each other).

Rank	Peak COM Excursion			MOS Variability		
	LASSO	F-Test	ReliefF	LASSO	F-Test	ReliefF
1	vAHS	vAHS	sAHS	sLHS *	vAHS	sAHS
2	sLHS *	sAHS	aAHS	sAHS *	vLHM	sAMD
3	sAHS *	vAMD	aAM	sAMD *	vLM	aAMD
4	sST *	vAM	aAMD	aLHM *	vLMD	sLHS
5	sAMD	vLM	sAM	aAMD *	vLHS	sAM
6	aLHM	sVHM	sAMD	sVHM *	vST	vLM30
7	aVHS	vLHM	aST	vLM30	vAMD	vAHS
8	aVHM	vST	aAM30	aVM30	sVHM	aAM
9	aAMD	vLM30	sLHS	vAHM	vVHM	sMD
10	sAM	sMD	vAHS	sMD	vAM	vM

**Table 4:** VIF values among top 10 features selected by each feature selection method for peak COM excursion and MOS variability (* indicates if VIF > 5).

Feature	Peak COM Excursion			MOS Variability		
	LASSO	F-Test	ReliefF	LASSO	F-Test	ReliefF
1	2.24	1.34	433.55 *	1.61	4.12	7.12 *
2	1.70	2.48	93.76 *	1.53	12.88 *	8.00 *
3	2.11	1.38	164.77 *	1.52	9.03 *	3.66
4	1.94	6.71 *	3.01	1.84	14.25 *	2.88
5	3.01	9.51 *	4.61	2.34	8.04 *	2.48
6	2.33	8.57 *	3.22	1.59	12.23 *	1.48
7	1.54	1.69	2.14	2.22	14.18 *	2.52
8	2.38	5.57 *	1.50	1.63	9.11 *	2.30
9	1.50	8.79 *	2.35	1.62	8.02 *	1.31
10	2.73	1.78	1.95	1.14	1.94	1.75

The names of the features for each model are shown in Table 3.

**Table 5:** Comparison of prediction error (MAE) for each feature selection method in peak COM excursion and MOS variability.

No. of Feature	Peak COM Excursion			MOS Variability		
	LASSO	F-Test	ReliefF	LASSO	F-Test	ReliefF
1	0.0997	-	-	-	-	-
2	-	0.0935	0.1007	-	0.0056	0.0061
3	-	0.0935	0.1006	-	0.0055	0.0051
4	0.0929	0.0936	0.1010	-	0.0056	0.0048
5	0.0916	0.0941	0.1007	-	0.0055	0.0049
6	0.0921	0.0947	0.0947	0.0045	0.0054	0.00474
7	**0.0883**	0.0901	0.0946	0.0045	0.0054	**0.00466**
8	0.0885	**0.0896**	0.0960	0.0044	0.0052	0.00468
9	0.0885	0.0899	0.0945	0.0043	0.0052	0.00469
10	0.0890	0.0901	**0.0909**	**0.0041**	**0.0051**	0.00472

The names of the features for each model are shown in Table 3.

**Table 6:** Results of the paired *t*-test and effect size between the actual and predicted values for peak COM excursion and MOS variability (* indicates *p* < 0.05).

Dependent Variable	Group	Mean	Standard Deviation	*p*-Value	Effect Size (Cohen’s *d*)
Peak COM excursion	Actual	0.3585	0.1513	0.0527	0.0053
	Predicted	0.3593	0.1041		
MOS variability	Actual	0.0215	0.0090	0.0318 *	0.0111
	Predicted	0.0216	0.0078		
